# Experimental and Computational Methods to Assess Central Nervous System Penetration of Small Molecules

**DOI:** 10.3390/molecules29061264

**Published:** 2024-03-13

**Authors:** Mayuri Gupta, Jun Feng, Govinda Bhisetti

**Affiliations:** 1 Department of Modeling and Informatics, Merck & Co., Inc., Rahway, NJ 07065, USA; 2 Department of Computational Chemistry, Cellarity, 101 South Street L6, Somerville, MA 02143, USA; jfeng@cellarity.com

**Keywords:** blood–brain barrier (BBB), CNS drug discovery, passive diffusion, active transport, efflux transporters, influx transporters, P-glycoproteins (P-gp), breast cancer resistance protein (BCRP), in silico models

## Abstract

In CNS drug discovery, the estimation of brain exposure to lead compounds is critical for their optimization. Compounds need to cross the blood–brain barrier (BBB) to reach the pharmacological targets in the CNS. The BBB is a complex system involving passive and active mechanisms of transport and efflux transporters such as P-glycoproteins (P-gp) and breast cancer resistance protein (BCRP), which play an essential role in CNS penetration of small molecules. Several in vivo, in vitro, and in silico methods are available to estimate human brain penetration. Preclinical species are used as in vivo models to understand unbound brain exposure by deriving the Kp,uu parameter and the brain/plasma ratio of exposure corrected with the plasma and brain free fraction. The MDCK-mdr1 (Madin Darby canine kidney cells transfected with the MDR1 gene encoding for the human P-gp) assay is the commonly used in vitro assay to estimate compound permeability and human efflux. The in silico methods to predict brain exposure, such as CNS MPO, CNS BBB scores, and various machine learning models, help save costs and speed up compound discovery and optimization at all stages. These methods enable the screening of virtual compounds, building of a CNS penetrable compounds library, and optimization of lead molecules for CNS penetration. Therefore, it is crucial to understand the reliability and ability of these methods to predict CNS penetration. We review the in silico, in vitro, and in vivo data and their correlation with each other, as well as assess published experimental and computational approaches to predict the BBB penetrability of compounds.

## 1. Introduction

**Figure 1:**
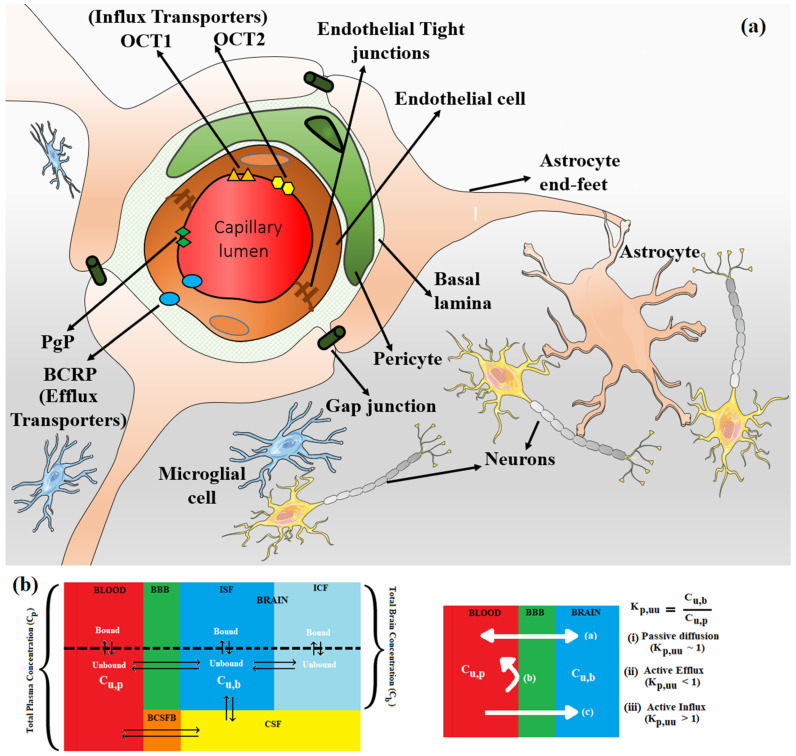
(**a**) The endothelial tight junctions of the BBB (shown in brown) severely restrict paracellular transport, whereas specialized transporters, e.g., P-gp (green diamond) and BCRP (blue oval) (efflux transporters) and OCT1 (orange triangle) and OCT2 (yellow hexagon) (influx transporters) regulate the transcellular transport of metabolic nutrients and other essential molecules across BBB. Enclosed in the basal lamina, pericyte cells partially surround these BBB endothelial cells. The complex tight cellular network of BBB is further maintained by astrocytes’ end-feet. Astrocytes maintain the cellular link between neurons and microglial cells. The transport across BBB involves concentration gradient-driven passive diffusion and active transport employing various efflux and influx transporters in the endothelial cell membrane. (**b**) Schematic of plasma and brain compartments presenting different modes of transport across BBB, i.e., passive diffusion and active transport using efflux (e.g., P-gp, BCRP) and influx (e.g., OCT1, OCT2) transporters. Kp,uu represents the unbound brain to unbound plasma drug concentration ratio, where Cu,b and Cu,p represent the unbound drug concentration in the brain and plasma, respectively. Different brain compartments, i.e., Blood, BBB, CSF, BCSFB, ISF, and ICF, correspond to blood, blood–brain barrier, cerebrospinal fluid, blood–cerebrospinal fluid barrier, interstitial fluid, and intracellular fluid, respectively.

In central nervous system (CNS) drug discovery, estimating the brain exposure to lead compounds is critical for their optimization. Compounds need to cross the blood–brain barrier (BBB) to reach the pharmacological targets in the CNS. The BBB is a complex physical barrier that surrounds most of the blood vessels in the brain and prevents the permeation of harmful molecules from circulating blood into the brain (see Figure 1a). The tight junctions of the BBB severely restrict paracellular transport, whereas specialized transporters, pumps, and receptors regulate the transcellular transport of metabolic nutrients and other essential molecules. Small lipophilic molecules can passively diffuse across the lipid bilayer but are often returned to the blood by efflux pumps [1,2]. Due to the impermeability of the CNS, it is a challenge for most molecules to gain access to the brain, although several molecules transfer from the blood to the brain.

Several mechanisms are potentially involved in this process [3]. While passive diffusion is a major mechanism of penetration of drugs into the CNS, efflux by several transporters such as P-glycoprotein (P-gp), breast cancer resistance protein (BCRP), and members of the multidrug resistance protein (MRP) family at the BBB limit concentration of drugs in the CNS [4,5,6]. Influx transporters such as OCT1 and OCT2 surrogate the penetration of bulky and charged molecules across the BBB (see Figure 1b). P-gp and BCRP are relatively well characterized among these efflux transporters, with considerable overlap among their substrates. P-gp (also known as MDR1 (multidrug resistance protein 1) and ABCB1 (ATP-binding cassette sub-family B member 1)) is widely expressed at the BBB. In the last 20 years, numerous studies conducted in P-gp knockout versus wild-type mice observed significant P-gp efflux of drugs [7]. Thus, the efflux of drugs by P-gp has been regarded as an essential factor determining the drug concentration in the brain. Further, over the past few decades, it has also become clear that reliance on total drug level in the brain is often misleading and that unbound drug concentration is more predictive of target occupancy and, ultimately, in vivo efficacy [8]. These developments led to the use of MDR1-MDCK in vitro assay to estimate the permeability and efflux of lead molecules and in vivo (rat or mouse) models to determine unbound brain exposure to lead molecules. 

A reliable in silico method for predicting the brain penetration of lead compounds would provide significant value and acceleration to drug discovery programs, save precious in vivo resources, and prioritize leads for in vivo assessment. The challenges faced in developing such in silico models arise from the complexity of the BBB, involving multiple transporters and the influence of multiple pharmacokinetic parameters. Also, the available datasets for training models are small and do not cover the entire drug space. Only a subset of compounds in these datasets has both in vitro and in vivo data. Nevertheless, prediction models are being built as they are crucial for assessing the CNS penetrability of compounds in commercial libraries, virtual libraries, and molecules generated by AI-enabled de novo design methods. Numerous in silico methods have been developed to predict brain exposure using different classification and/or regression algorithms. The type of experimental data used to build prediction models changed from the simple classification of BBB+ (for penetrating compounds) and BBB− (for non-penetrating compounds) to Kp (logBB, logarithm of the ratio of total steady-state concentration in the brain to that in blood at a given time) to more recent Kp,uu (the unbound brain-to-unbound plasma concentration ratio). In many publications, the easily accessible and abundant classification data (BBB+ for penetrating and BBB− for non-penetrating compounds), often estimated by the presence or absence of CNS activity, are used. Earlier QSAR and machine learning prediction models utilized logBB and logPS (logarithm of the permeability surface area product) data. LogBB lacks information regarding the free drug concentration available for transport across the BBB, and logPS does not incorporate BBB transporter-mediated efflux. These models were extensively reviewed elsewhere [9] and are not discussed in this review as there has been a paradigm shift away from optimizing Kp toward Kp,uu in CNS drug discovery. Free tissue drug concentration, Kp,uu, is considered to be the therapeutically relevant metric for estimating free drug concentration at the receptor site over the time course of its action, not the total drug concentration, Kp, based on the free drug hypothesis [8,10,11]. From a compartmentalized CNS drug distribution model (see Figure 1b), steady-state Kp,uu can be presented in terms of passive diffusion (PassDiff), active influx clearance (${Cl}_{influx}$), active efflux clearance (${Cl}_{efflux}$), brain interstitial fluid bulk flow clearance (${Cl}_{bulk}$), and brain metabolic clearance (${Cl}_{metabolism}$). ${Cl}_{bulk}$ and ${Cl}_{metabolism}$ become insignificant and can be disregarded for molecules with high permeability and low metabolic clearance. The Kp,uu parameter presents efflux and influx permeation across the BBB relative to passive diffusion. Passive diffusion, active efflux, and active influx correspond to values of unity, below unity, and above unity, respectively. Kp,uu can be understood as a measure of lateral efficacy of various efflux and influx transporters independent of the extent of brain or plasma tissue binding (Equation (1)).

(1) $$K_{p,uu}=\frac{PassDiff+{Cl}_{influx}}{PassDiff+{Cl}_{efflux}+{Cl}_{bulk}+{Cl}_{metabolism}}$$

The closer the Kp,uu value is to 1, the less peripheral body burden is required to achieve efficacious free concentration in the brain. Generally, a Kp,uu > 0.3 in rats is considered adequate, although this value depends on the drug’s potency and other ADME properties. Therefore, this review will focus on the models that utilize the preclinical in vivo Kp,uu data and the MDR1-MDCK in vitro data in validation and training sets, as well as the physicochemical properties, different multiparameter scores, and the prediction models that distinguish CNS and non-CNS drugs. The applicability and limitations of different in silico methods will also be discussed.

## 2. Physicochemical Properties of CNS Drugs

Despite significant challenges in designing compounds that cross the BBB, multiple classes of drugs cross the BBB as they are known to treat CNS diseases, and many more CNS drugs are in clinical development [12]. The principle medicinal chemistry strategy in drug discovery has been to optimize a compound’s physicochemical properties to maximize its CNS penetration.

Since the publication of Lipinski’s rule of 5 in 1997 that defined desirable physicochemical properties (MW < 500 Da, log P < 5, HBD < 5, and HBA < 10) for oral bioavailability of a drug candidate [13], several groups attempted to map the physicochemical space of CNS drugs employing different approaches. Hansch et al. [14] studied a dataset of 201 barbiturates with preclinical in vivo efficacy data and found the in vivo efficacy of the drug to have a parabolic dependency on logP and suggested a logP value of 2 for optimal in vivo activity. The improved chance of CNS penetration was predicted for the following desirability ranges: MW < 450, PSA < 90 Å^2^, and Log D [1, 4] for a dataset of 125 CNS and non-CNS drugs analyzed by Van der Waterbeemd et al. [15]. In a study of 776 CNS and 1590 non-CNS oral drugs that reached at least phase 2 clinical trials, Kelder et al. [16] suggested an upper polar molecular surface area (PSA) limit of <60–70 Å^2^ for most CNS drugs. Doan et al. [17] have indicated that physiochemical properties of CNS drugs differ substantially from non-CNS drugs, with CNS dataset mean values of cLogP (3.43), cLogD (2.08), HBD (0.67), and PSA (40.5 Å^2^) for a dataset containing 48 CNS and 45 non-CNS drugs. Norinder et al. [18] suggested that a molecule where O + N < 5, or cLogP − (O + N) > 0, has an improved chance of CNS penetration. The physiochemical property space suggested by Didziapetris et al. [19] for better CNS penetration while avoiding P-gp efflux liability had the following parameters: MW <400, pKa <8, and N + O < 4. Leeson et al. [20] suggested mean values of 310 (MW), 4.32 (O + N), 2.12 (HBA), and 4.7 (RB) for CNS drug molecules based on a review of a dataset of 329 oral drugs marketed between 1983 and 2002. The recommended attributes of successful CNS drugs suggested by Pajouhesh et al. [12] for a study of a dataset of marketed CNS drugs were MW < 450; H-bonds < 8; pKa 7.5−10.5; HBD < 3; HBA < 7; RB < 8; cLogP < 5; and PSA < 60−70 Å^2^. Based on a medicinal chemistry literature review, Hitchcock et al. [21] recommended physicochemical property ranges for improving BBB penetration: MW < 500, PSA < 90 Å^2^, cLogD (pH 7.4) [2, 5], cLogP [2, 5], and HBD < 3.

It was realized that CNS drugs occupy considerably smaller chemical space than oral drugs designed for peripheral targets [22]. Indeed, CNS drugs tend to be smaller with higher lipophilicity and lower polar surface area (PSA) than non-CNS drugs (see Table 1).

In the broadest sense, moderately lipophilic drugs cross the BBB by passive diffusion, and the hydrogen bonding properties of drugs can significantly influence their CNS uptake profiles. Polar molecules are generally poor CNS agents unless they undergo active transport across the CNS. Size, ionization properties, and molecular flexibility are other factors observed to influence the transport of a compound across the BBB. The design of CNS drug candidates with intracellular targets may benefit from increased basicity and/or the number of hydrogen bond donors [23]. However, most of the “older” CNS drugs with common pharmacophoric features of small lipophilic amines were modulators of ion channels, monoamine GPCR, and transporters [24]. This scenario is rapidly changing as CNS drug discovery efforts have been shifting towards emerging therapeutic areas such as neurodegeneration and neuro-oncology with novel “non-traditional” CNS targets. The compounds targeting these new targets are relatively larger and more polar with wider chemical diversity. It is, therefore, possible that the current understanding of the allowed physicochemical properties space of CNS drugs will expand. 

## 3. BBB Penetration Scoring Schemes for Predicting Brain Penetrance across the BBB Primarily by Passive Diffusion

Analyzing the physicochemical properties of CNS (CNS+) and non-CNS (CNS−) drugs led to the formulation of different scoring schemes to design CNS drugs. Recently, multiple algorithms have been proposed to improve RO5 for drug discovery in the CNS target space. Wager et al. [25,26] developed an algorithm called “multiparameter optimization (MPO)” based on a study of 119 CNS drugs and 108 CNS clinical candidates to suggest the optimal range of property space for different physicochemical properties of drug molecules. For each of these calculated properties, a range of values is identified as more favorable (score = 1) or less favorable (score = 0) for a CNS drug candidate. The algorithm comprises six physiochemical properties with median values for CNS drugs: MW 305.3 Da, PSA 44.8 Å^2^, HBD = 1, cLogP 2.8, cLogD 1.7, and pKa = 8.4. This scoring method showed that 74% of marketed CNS drugs and Pfizer CNS drug candidates displayed a high CNS MPO score (MPO desirability score ≥ 4, using a scale of 0–6). However, a follow-up study involving re-examining the MPO score by the authors suggested that the MPO score can vary substantially depending on the computational software and method used to calculate physiochemical properties (mainly LogD and pKa) [27]. Also, the MPO score is congenitally biased toward lipophilicity parameters. The MPO score also poses the risk of populating the chemical space with small molecules with very low molecular weights, as the MPO score does not apply lower limits (e.g., clogP, clogD, MW, and pKa) but only applies upper limits to physiochemical properties used. These very-low-molecular-weight small molecules may not bind to certain targets of interest with sufficient binding potency. The MPO score also does not characterize non-CNS drugs, as it is trained on the dataset of CNS drugs (119) and CNS drug candidates (108). A separate study assessing 616 compounds with measured unbound concentrations in the brain confirmed that a higher CNS MPO score correlated with a higher unbound concentration in the brain [22]. A probabilistic MPO scoring function, designated as pMPO, is based on defining the physiochemical properties of a dataset of 299 CNS-penetrant and 366 non-CNS-penetrant molecules. pMPO physiochemical descriptors, along with their weighting, are as follows: TPSA (0.33), HBD (0.27), MW (0.16), clogD (0.13), and basic pKa (0.12) [28]. Ghose et al. [29] studied a dataset of 317 CNS and 626 non-CNS oral drugs and have proposed property ranges for CNS penetration: TPSA < 76 Å^2^ (25−60 Å^2^), 740−970 Å^3^ volume, N [1, 2], linear chains outside of rings < 7 [2, 4], HBD < 3, (0,1), and SAS (460−580) Å^2^; the ranges given in the parentheses are preferred. They optimized the relative weights of these parameters by comparing the physicochemical property distribution of CNS versus non-CNS oral drugs. Rankovic et al. [30] mapped the physiochemical properties of a diverse corporate dataset from Eli Lilly based on brain-penetrant and peripherally confined molecules. They developed an algorithm termed MPO_V2, which contained five descriptors. They dropped the LogD descriptor from MPO and included double weight for the HBD descriptor (MPO_V2: ∑T0 (clogP, MW, TPSA, pKa, and 2 HBD). However, this article was retracted [30]. Recently, Gupta et al. [31] proposed the Blood−Brain Barrier (BBB) Score that is composed of stepwise and polynomial piecewise functions with five physicochemical descriptors: number of aromatic rings, heavy atoms, MWHBN (a descriptor comprising molecular weight, hydrogen bond donor, and hydrogen bond acceptors), topological polar surface area, and pKa. The BBB Score outperformed (AUC = 0.86) the CNS MPO approach (AUC = 0.61).

**Figure 2:**
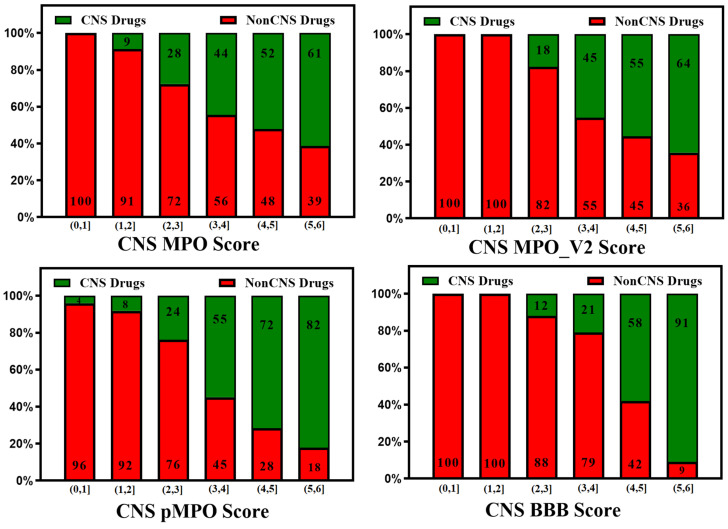
Relative distribution of CNS class of compounds: CNS+ (green) and CNS− (red). The MPO, MPO_V2, pMPO, and BBB scores range from 0 to 6 (a score within a range of [4, 6] means better CNS penetration). Original pMPO scores range between 0 and 1. To be consistent with MPO scores, we scaled the pMPO scores from 0 to 6.

**Figure 3:**
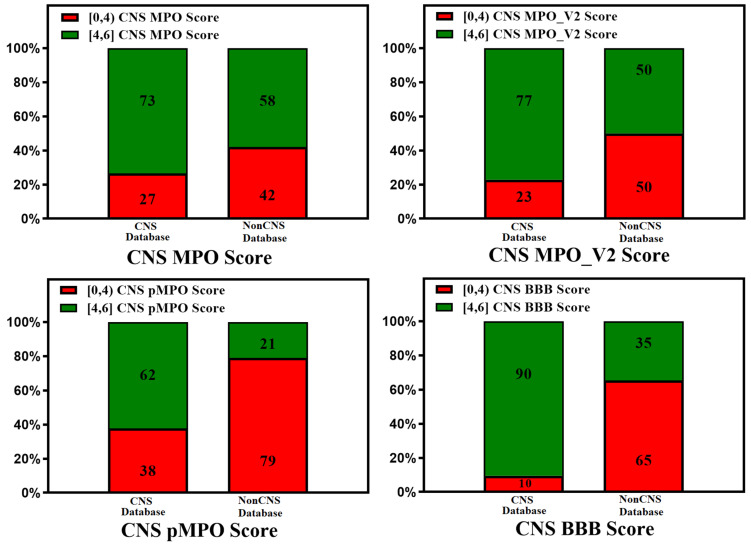
Relative distribution of CNS class of compounds: CNS+ (green) and CNS− (red). The MPO, MPO_V2, pMPO, and BBB scores range from 0 to 6 (a score in the range of [4, 6] means better CNS penetration). Original pMPO scores range between 0 and 1. To be consistent with MPO scores, we scaled the pMPO scores from 0 to 6. Percentage of CNS drugs and non-CNS drugs correctly identified (for CNS: MPO, MPO_V2, pMPO, BBB Score (4, 6]; for non-CNS: MPO, MPO_V2, BBB Scores [0,4)) in their respective CNS and non-CNS database is plotted on 100% stacked bar graph for MPO, MPO_V2, pMPO, and BBB Scores.

The ease of calculation of the CNS MPO, CNS MPO_V2, CNS pMPO, and CNS BBB scores and their capability to predict the BBB penetration of compounds can aid in mapping the property space of large commercial and virtual compound libraries to support lead optimization. CNS MPO and CNS BBB scores are widely utilized for CNS drug discovery programs. Figure 2 and Figure 3 represent 100% stacked bar graphs for low to high CNS MPO, MPO_V2, pMPO, and BBB scores for a dataset of CNS and non-CNS drugs. Ideally, a CNS MPO, MPO_V2, and BBB score in the range of (4,6] should correlate to a CNS drug, and a CNS MPO, MPO_V2, and BBB score of (0,4] should correspond to a non-CNS drug. pMPO outputs a score in the range of (0,1], which has been scaled to (0,6] to compare against other scores. However, the CNS BBB score performs better in identifying a higher percentage of CNS compounds than other scores.

In addition to scoring methods, quantitative structure–activity relationship (QSAR) and machine learning (ML) algorithms were successfully applied to predict BBB permeability. The derivation of QSAR and ML models involves calculating molecular descriptors, fitting them to experimental values using a statistical algorithm on a training dataset, and predicting experimental values of the test dataset. Various QSAR and ML methods such as support vector machine (SVM), decision tree (DT), and k-nearest neighbor (KNN) that combine property-based descriptors with molecular fingerprints of compounds predict the classification of CNS and non-CNS drugs with reasonable accuracy [32,33,34,35]. Chen et al. [36] and Varadharajan et al. [37] employed machine learning algorithms (random forest (RF) and support vector machine (SVM)) to develop direct and indirect regression models. For 173 compounds in the training set [36], their model used a total of 196 descriptors. Their model predicted the Kappa2 descriptor, i.e., the measure of molecular linearity being strongly correlated while having poor reliability on the lipophilicity of a molecule, as predicted by Fridén et al. [38]. Similarly, Saxena et al. [39] published accurate classification models with different ML algorithms using physicochemical properties, Molecular ACCess Systems keys fingerprint (MACCS) [40], and substructure fingerprints. A deep learning (DL) method achieved better accuracy than the ML methods on three different datasets [41]. Recently, various DL methods such as artificial neural networks (ANN), deep neural networks (DNN), convolutional neural networks (CNN), recurrent neural networks (RNN), and graph convolutional neural networks (GCNN) have been used to predict BBB permeability with high accuracy [42,43,44,45,46,47,48]. Alsenan et al. [49] published a highly accurate deep learning model based on a recurrent neural network. Zhang and Ding [50] deployed SVM and Greedy Algorithms to identify key features of CNS drugs. Shaker et al. [51] used the light gradient boost machine (LightGBM) algorithm to develop the LightBBB server (http://ssbio.cau.ac.kr/software/bbb; accessed on 16 January 2024) to predict BBB permeability. An AUC of 0.93 was achieved for a large dataset of 7162 compounds using 10-fold cross-validation. Parakkal et al. [52] employed a mixed deep learning model on the same dataset and achieved slightly better accuracy (AUC = 0.96, accuracy = 92%). Recently developed machine learning- and deep learning-based ADMET predictors, including BBB permeation prediction, with reasonable accuracy (mostly AUC~0.90) are hosted on the web: AdmetSAR2.0 [53], ADMETLab2.0 [54], FP-ADMET [55], Interpretable-ADMET [56], DeepB^3^ [57] and HelixADMET [58]. In spite of the good accuracy of these models, their lack of interpretability limits their use in designing and optimizing CNS drugs. Tong et al. [59] showed the utility of uncertainty estimations for improving the predictability of deep learning BBB permeability models. An excellent review of classification models using different datasets and different ML and DL algorithms is published by Saxena et al. [60] and Tran et al. [61]. These qualitative classification models are helpful for quick screening of large compound databases at early-stage drug discovery.

## 4. Active Transport across the BBB (Efflux Transporters, Influx Transporters, and Kp,uu)

Kp,uu (the unbound brain-to-unbound plasma concentration ratio) is an important parameter to estimate the unidirectional or bidirectional active transport of drugs across the BBB via specified influx and efflux transporters. As discussed in Equation (1) above, Kp,uu presents a measure of lateral efficacy of various efflux and influx transporters independent of the extent of brain or plasma tissue binding. Quantitative prediction of Kp,uu by QSAR and ML methods has been challenging [62,63]. The limited size of the training sets of compounds combined with highly variable (fivefold) experimental Kp,uu data is the main reason for the moderate performance of the models. Three experimental techniques are usually employed to estimate experimental Kp,uu: (a) microdialysis, (b) brain homogenate, and (c) the brain slice method. Each method has advantages and challenges; the variability within experimental results exists even within the same method based on the different experimental setups, preclinical species, and protocols. A detection probe is surgically implanted into the brain to estimate the unbound concentration of a molecule in the microdialysis method, which is considered an in vivo gold standard for measuring Kp,uu [64,65,66]. However, this method poses many technical challenges, including recovery of the microdialysis probe while working with lipophilic drugs, a large number of resources, time demands, risk of brain injury, and associated increase in BBB permeability; additionally, it necessitates the use of lots of animals, leading to ethical concerns and making it less applicable in the initial phases of drug discovery [67,68,69]. The brain homogenate method introduced by Kalvass et al. [70] involves dialyzing a small sample of brain homogenate infused with the molecule into a 96-well equilibrium dialysis apparatus. This method is used for high-throughput screening of CNS drugs; unbound brain concentration is calculated from total steady-state brain concentration and free fraction [71]. One drawback of this method is that the binding properties of brain tissue could be changed during brain homogenization, which unfolds new binding sites and results in the underrepresentation of available free fractions [72]. In this review, we have compiled Kp,uu values from Summerfield et al. [73,74] and Culot et al. [75]. In the brain slice method of calculating Kp,uu, animal brain slices (usually rat or mouse) are infused with test molecules incubated at 37 °C in either plasma or buffer solution. The test amount of buffer or plasma solution at designed time points is withdrawn. Kp,uu is calculated as a ratio of in vivo total brain-to-plasma concentration (Kp) and in vitro unbound brain volume of distribution (Vu, brain) and the unbound fraction of drug in plasma (fu, plasma) in the incubated brain slices. The brain slice method has the advantage over other methods as the cell structure in brain tissue is maintained in brain slices, and this method could be developed as a high-throughput screening method [72,76,77]. Recently, Langthaler et al. [78] presented mini pig brain penetration data (Kp,uu) for a reference set of 17 compounds with varying physiochemical properties. Loryan et al. [79] reviewed the importance and impact of the Kp,uu,brain concept in contemporary drug discovery. They concluded that Kp,uu is the gold standard for studying CNS penetration, but its impact on CNS drug discovery has been limited so far due to several factors such as (a) costly and time-consuming experimental procedures and (b) a lack of Kp,uu data in higher species (e.g., monkeys, pigs).

The lack of experimental data for Kp,uu has also limited the development of highly accurate in silico prediction models of Kp,uu. Only a few in silico models of Kp,uu with moderate accuracy have been reported [23,36,37,38,80,81,82,83,84,85,86,87,88]. Poor performance of global models of Kp,uu is understandable as (a) the training datasets do not have good coverage of chemical space, and (b) models need to account for multiple factors that affect brain penetration, e.g., experimental protocols and animal species, as explained above. In some cases, the higher-than-expected accuracy reported for some of these ML models may be due to model overfitting [89]. Two QSAR models of Kp,uu employing the PLS method for a training set of 41 marketed drugs with experimental Kp,uu have been developed by Fridén et al. [38]. The first model used 16 molecular descriptors with an R^2^ of 0.45 and the second one achieved a similar accuracy with an R^2^ of 0.43 using only a single descriptor of a number of hydrogen bond acceptors. This comparative study demonstrates the predominant role of hydrogen bond acceptors in determining a compound’s CNS permeability. An indirect regression model using the same data showed reasonable accuracy with an R^2^ of 0.74 [80] but performed poorly when validated against the dataset published by Summerfield et al. [43]. Loryan et al. [23] trained a PLS regression QSAR model on a dataset of 39 Kp,uu values using two molecular descriptors (vsurf_Cw8 and TPSA) with moderate accuracy (R^2^ = 0.82 and RMSE = 0.31). However, this model’s performance was unsatisfactory when validated against the Fridén et al. [38] dataset. Zhang et al. [82] developed a binary Kp,uu classification model on a dataset containing 677 and 169 molecules in training and test sets, respectively, which showed similar accuracy (R^2^~0.75). Lawrenz et al. [83] proposed a physics-based approach to predict Kp,uu using solvation energy from quantum mechanical calculations. 

**Figure 4:**
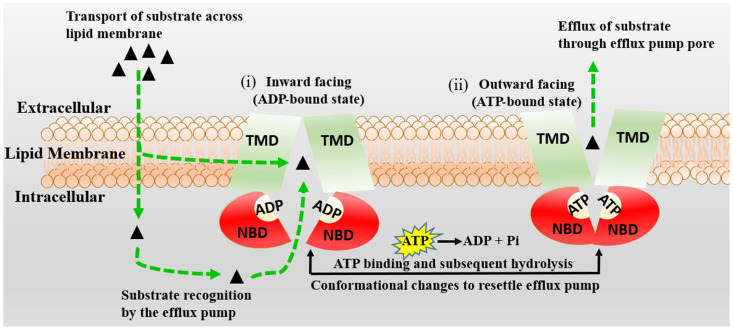
The schematic diagram of the proposed mechanism of P-gp (MDR1) is represented. Transmembrane (TBDs) and nucleotide (NBDs) binding domains of P-gp are presented in green and red, respectively. The P-gp substrates are shown by black triangles, which cross the BBB membrane by passive diffusion or active transport. The inward-facing, ADP-bound state structure (**i**) changes conformation, the NBDs dimerize, and the TMDs re-orientate to extracellular space to adopt an outward-facing (ATP-bound) state (**ii**). The extracellular segment’s transmembrane helices in the outward-facing conformation of P-gp reorient to release the substrate. Upon ATP hydrolysis, the transporter is reoriented to the inward-facing structure, and two phosphate molecules are released.

Integrating Kp,uu in silico models with the knowledge of molecule interactions in BBB efflux and influx transporters [90], which influence brain permeability, can improve Kp,uu model performance [63]. Of the many influencing factors such as active uptake, brain metabolism, bulk flow, passive permeability, etc., efflux by several membrane transporters, such as P-gp, has a dominant role [82,91]. Langthaler et al. [78] have also reported in their recent study that the most prominent difference in Kp,uu across different species (mini pig, dog, monkey, human) was observed for transporter substrates. Rodent transporter knockout models have also been employed to assess brain penetration in drug discovery [92]. P-gp is widely expressed at the BBB. It transports molecules against a concentration gradient, utilizing the energy of ATP hydrolysis (See Figure 4).

**Figure 5:**
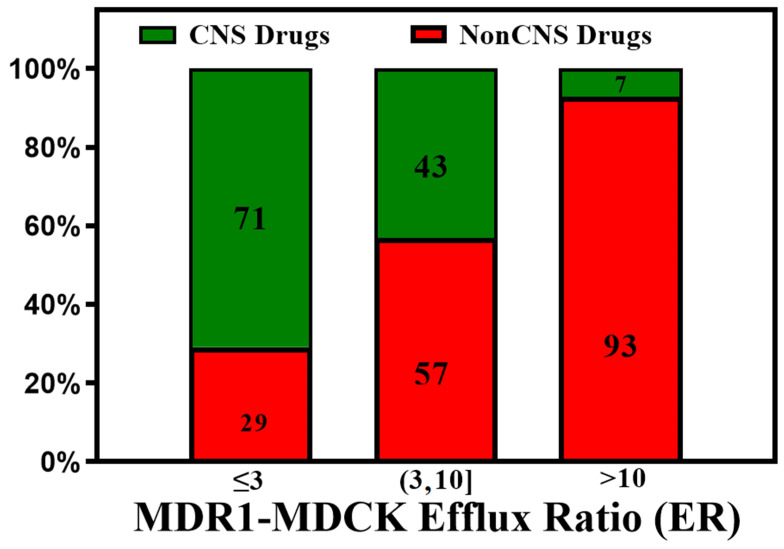
A plot of the CNS class of compounds CNS+ (green) and CNS− (red) against their measured efflux ratios. CNS+ compounds with good brain exposure have a higher probability of having lower efflux.

Dolgikh et al. [81] incorporated the P-gp efflux ratio in direct and indirect regression QSAR models for Kp,uu. The performance of the Kp,uu model improved significantly by adding P-gp efflux data (R^2^ enhanced from 0.39 to 0.53). For understanding the quantitative correlations between the structure of P-gp and the various molecular descriptors, different computational algorithms accompanying structure- and ligand-based approaches [93,94], pharmacophore models [95,96], and machine learning methods [97] have been studied. With the availability of high-throughput P-gp efflux data using MDCK-MDR1 assays, there is an increasing effort to measure the efflux of a large number of compounds experimentally [98]. These data enabled better predictive models for P-gp efflux. Ohashi et al. [99] constructed regression models to predict the value of P-gp-mediated efflux using 2397 data entries with an extensive dataset collected under the same experimental conditions. Most compounds in the test set fell within two- and three-fold errors in the random forest regression model. P-gp transporter efflux data available in the literature have considerable variability, as molecules are tested using different protocols, cell lines, and biological assays [100]. Broccatelli et al. [101] tested a dataset of 187 compounds in the Borst-derived MDCK-MDR1 cell lines to calculate P-gp efflux ratios (ERs). The ER presents the ratio of the apparent permeability from the basolateral to the apical direction (excretory) to the apparent permeability from the apical to the basolateral direction (intake) in an overexpressing P-gp cell line. Molecules with an ER ≥ 2 are typically considered P-gp substrates [102]. Available P-gp efflux data (measured in MDCK-MDR1 cells) for CNS (CNS+) and non-CNS (CNS−) drugs show that most CNS drugs have a P-gp efflux ratio below 10. In cases where brain metabolism and uptake effects are negligible, it has been shown that compounds with higher efflux generally have lower Kp,uu values. (See Figure 5). As CNS distribution of a compound does not depend only on P-gp efflux, a significant percentage of compounds with lower efflux do not distribute into the CNS, and a good percentage of compounds with higher efflux distribute into the CNS. It is important to note that the analysis involves a small number of drugs (128 CNS+ and 39 CNS−) that have MDR1-MDCK efflux data and provide qualitative guidance to utilize efflux data for selecting CNS-penetrant compounds.

These efflux transporters modulate the brain exposure to a drug without affecting systemic exposure. It was recognized that passive permeability and P-gp efflux impact the extrusion of drugs from the brain and that in vitro efflux ratios (ER) can predict in vivo brain penetration [17,103,104]. Incorporating in vitro P-gp efflux information into the computational models improved the predictive performance of a QSAR model, as explained above [81]. Recently, Gupta et al. [88] have augmented the previous Kp,uu models by incorporating the role of various efflux and influx transporters. This algorithm gives the Brain Exposure Efficiency (BEE) Score and is devised based on a comprehensive series of QSAR calculations and molecular modeling simulations and implemented as an open-source calculator for predicting the unidirectional or bidirectional active transport of molecules across the BBB via specified transporter proteins. The model performed better with the Kp,uu data obtained from the brain slice method than with the Kp,uu data obtained from the microdialysis or brain homogenate methods [88]. The BEE score is implemented in MOE software as an SVL utility to predict Kp,uu, and Cu,b (unbound concentration of the molecule in the brain). More recently, Kosugi et al. [105] reported improvement in the predictivity and coverage of application by machine learning approaches for Kp,uu prediction by incorporating in vitro P-gp and BCRP activities.

**Figure 6:**
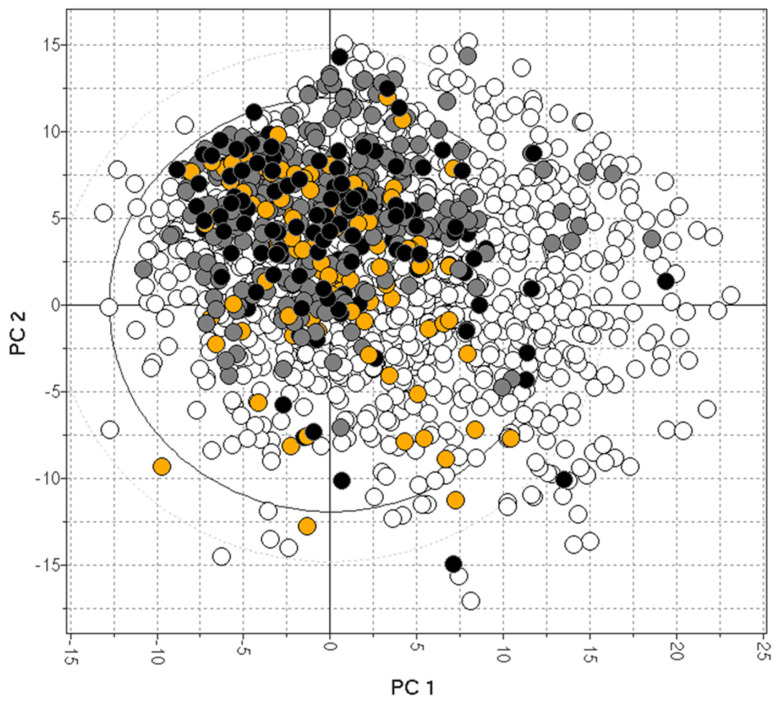
The bias of available drugs with MDCK (orange) and Kp,uu data (black). The grey and white dots represent CNS+ and CNS- compounds, respectively.

In vitro MDR1-MDCK represents a valid assay for predicting human P-gp efflux. It generally correlates well with the in vivo Kp,uu of preclinical species, although other transporters (like BCRP) may cause a disconnect. Consistent Kp,uu and P-gp efflux data are available for only a limited number of drugs [38,74,88,101,106] and are plotted in Figure 6, showing the limited data coverage of the drug space [82].

## 5. In Silico, In Vitro, and In Vivo Correlations

**Figure 7:**
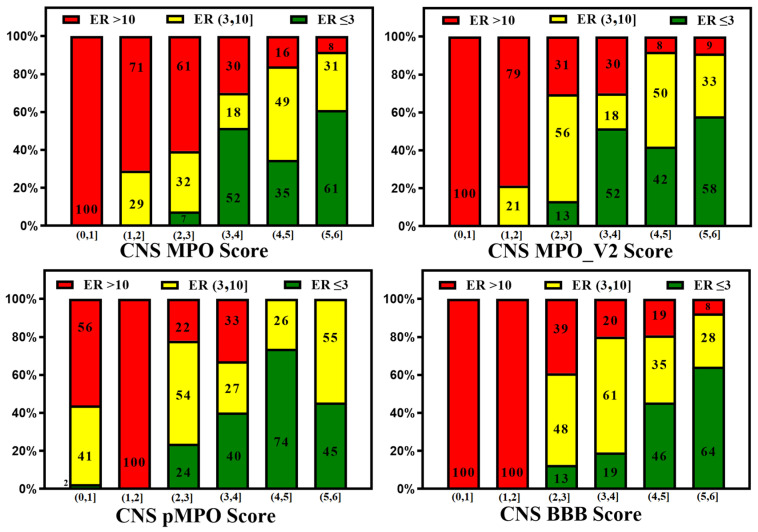
In silico methods (CNS MPO, MPO_V2, pMPO, and BBB Score) segregate low vs. high efflux compounds, but there is much room for improvement.

**Figure 8:**
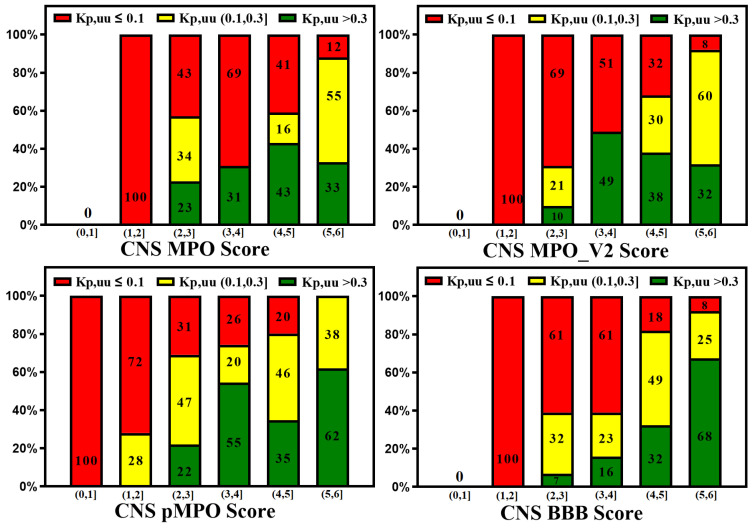
100% stacked bar graphs for low-to-high MPO, MPO_V2, pMPO, and BBB Scores for rat Kp,uu dataset. Compounds with a higher score tend to show higher unbound brain exposure.

A reliable in silico prediction method for CNS penetration can provide several advantages for discovering small-molecule drugs for neurological diseases but only if prediction results correlate with in vitro and in vivo measurements. Various CNS scoring schemes are fast and easy to apply to screen libraries at the exploratory stage of drug discovery; these scores correlate to some extent with in vitro efflux and animal Kp,uu, as illustrated by Figure 7 and Figure 8, which plot the relative distribution of CNS and non-CNS drugs based on the P-gp efflux ratio and rat Kp,uu corresponding to different CNS scoring schemes (CNS MPO, MPO_V2, pMPO, and BBB Score). Ideally, CNS drugs, i.e., the range of scores (4,6], should be more populated with drugs having an ER ≤ 3, and non-CNS drugs should be heavily populated in the lower ranges of scores, i.e., (0,4]. It is encouraging to see in Figure 7 that all scoring methods segregate the drugs with lower ER towards a higher score range (4,6], and the drugs with higher ER towards a lower range (0,3}, but the segregation is not perfect. There is a lot of room for improvement. Figure 8 presents 100% stacked bar graphs for low-to-high MPO, MPO_V2, pMPO, and BBB Scores for the rat Kp,uu dataset. Ideally, any molecule with an MPO, MPO_V2, pMPO, and BBB Score in the range of (4,6] should correspond to a CNS drug. Most CNS drugs have Kp,uu in a moderate range (i.e., Kp,uu of (0.1,0.3] or <0.3). Ideally, the in silico scores plotted in Figure 8 could be interpreted as the probability of a molecule attaining a score between 4 and 6 to have decent Kp,uu. MPO and MPO_V2 predict 33% and 32% of drugs to have a Kp,uu > 0.3, respectively, in the range (5,6], which is very low compared to the 62% and 68% predicted by the pMPO and BBB Score, respectively. For MPO_V2 and pMPO, drugs in the range (3,4] have poor predictability (nearly 50%) in differentiating between high and low Kp,uu exposure. The MPO and BBB Score predict that 69% and 61% of drugs that score in the (3,4] range have a Kp,uu ≤ 0.1, which is an improvement over the MPO_V2 and pMPO scores.

**Figure 9:**
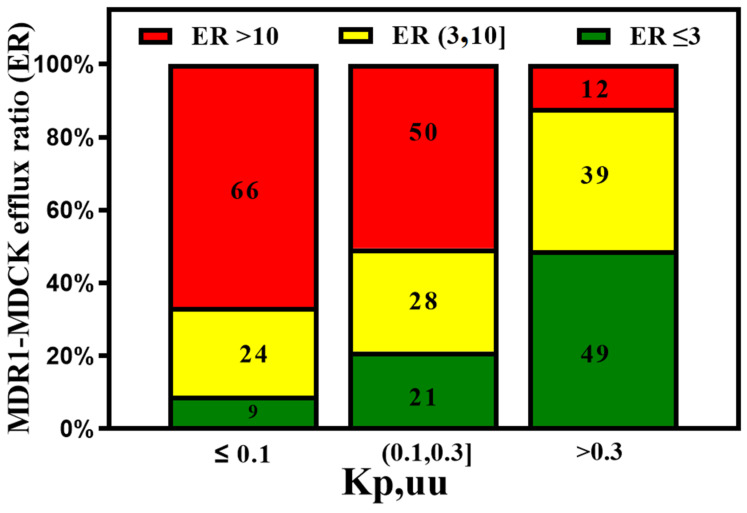
The MDR1-MDCK in vitro assay predicts good in vivo Kp,uu when ER < 3. However, compounds with medium efflux (3,10] also show moderate in vivo brain exposure.

On the other hand, the efflux ratio correlates well with animal Kp,uu (Figure 9).

**Figure 10:**
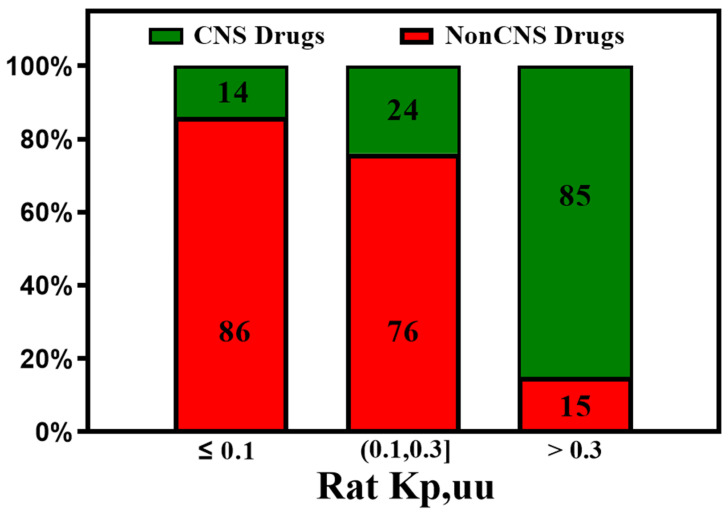
The Kp,uu of preclinical species is an important parameter for predicting human brain exposure. Most of the CNS drugs show rat Kp,uu over 0.3.

Similarly, high rat Kp,uu correlates well with human BBB penetrability. Zhang et al. [82] found the vast majority (>85%) of the CNS drugs show a rat Kp,uu over 0.3, which is consistent with our analysis of the available data shown in Figure 10.

Animal Kp,uu measurements are also nontrivial, expensive, and time-consuming. Such measurements are made for compounds at the lead optimization of drug discovery. On the other hand, in vitro measurements are faster, less expensive, and utilized extensively at earlier stages of discovery. In silico methods that rely only on chemical structure information (like the novel Kp,uu prediction method proposed by Watanabe et al. [107]) are highly useful at the Hit identification and Hit expansion stages. 

The ultimate purpose of predictive models is to improve the odds of success of drug candidates for CNS diseases. Patel et al. [108] outline a parallel analysis of previously published models for predicting brain penetration that utilizes MDR1-MDCK efflux data as a better predictor of brain penetration. They demonstrate the ability to harness lower species’ preclinical data to predict human brain availability. Sato et al. [109] described a translational CNS steady-state drug disposition model to predict Kp,uu across rats, monkeys, and humans using only in vitro and physicochemical data. This model can potentially minimize animal use and speed up CNS drug discovery.

## 6. Methods

**Dataset collection:** The first step involved collecting a diversified dataset of CNS and non-CNS drugs from different sources. A dataset of pre-1983 (864) and 1983−2002 (329) orally administered drugs was assembled from supporting information given by Leeson et al. [20] An FDA-approved drug library containing 3067 compounds and a CNS-penetrant compound library containing 221 compounds were obtained from https://www.selleckchem.com/screening/fda-approved-drug-library.html (accessed on 16 January 2024) and https://www.selleckchem.com/screening/cns-penetrant-compound-library.html (accessed on 16 January 2024), respectively, along with their development status, indication, SMILES, and physiochemical property data. Further information about drug structures, Kp,uu, and PgP data was taken from DrugBank (http://www.drugbank.ca/ (accessed on 16 January 2024)). CNS and non-CNS drug molecules were also obtained from different literature sources, including Rankovic et al. [30] and Wager et al. [26]. Literature Kp,uu data are compiled from three experimental techniques: microdialysis [67,68,69], brain slice [72,77], and brain homogenate [70,71] for only one animal species (rats) with an intravenous infusion administration method. The dataset of P-gp efflux ratios (ERs) in the MDCK-MDR1 cell line was compiled from Broccatelli et al. [101].

**Data Curation:** CNS and non-CNS drug datasets were merged from different sources. After removing the duplicates, the initial classification of CNS and non-CNS drugs was conducted based on the “drug category”, “indication”, “mechanism of action”, and “pharmacodynamics” fields in DrugBank. Other sources, e.g., SciFinder (https://www.cas.org/solutions/cas-scifinder-discovery-platform/cas-scifinder-n (accessed on 16 January 2024)), Drugs@FDA (http://www.accessdata.fda.gov/scripts/cder/drugsatfda (accessed on 16 January 2024)), and Pharmacogenomics Knowledge Base (http://www.pharmgkb.org (accessed on 16 January 2024)) were referenced to ensure correct classification of CNS and non-CNS drugs. Non-CNS drugs showing CNS side effects at standard therapeutic doses and CNS drugs that cross the BBB through active transport were manually removed from non-CNS and CNS drug datasets, respectively. We aimed to have the CNS drug dataset only contain drugs with passive diffusion across the BBB and the non-CNS drug dataset to be free of any drugs documented to have CNS side effects. The curated CNS and non-CNS datasets contained 320 and 770 molecules, respectively.

**Performance evaluation of different in silico models for curated datasets:** For the curated CNS and non-CNS datasets, we calculated scores using various in silico BBB permeation algorithms (CNS MPO [25,26], MPO_V2 [30], pMPO [28], and BBB Score [31]) and carried out their performance evaluation compared to each other. These predictions were further assessed for their correlation against experimental Kp,uu and Pgp ER data from the literature [38,73,74,75,101]. The MPO, MPO_V2, pMPO, and BBB scores range from 0 to 6 (a score in the range of (4,6] means better CNS penetration). Original pMPO scores have a range of 0 to 1. To be consistent with MPO scores, we scaled the pMPO scores from 0 to 6.

## 7. Conclusions

The successful discovery of small-molecule drugs to treat neurological disorders requires CNS penetration of these drugs [110]. The design of CNS-penetrable molecules was initially guided by the physicochemical properties of a limited set of known CNS active drugs and drugs that cause side effects in the CNS. The CNS MPO scores and later improvements were useful in simultaneously optimizing multiple properties of molecules for CNS targets. However, these scores do not always correlate well with in vitro (P-gp efflux) and animal in vivo (Kp,uu) data. The machine learning and deep learning models that classify CNS and non-CNS drugs achieve higher accuracy and are successfully applied to screen vast chemical libraries in the early phase of drug discovery but are found to be less beneficial for lead optimization. The need for higher accuracy in silico models with broader applicability is recognized, but such efforts require much more in vitro and in vivo data. These findings have prompted efforts to generate in vivo animal Kp,uu data and in vitro efflux data on a large number of compounds covering the available drug-like molecule space. Biogen scientists recently released in vitro efflux data on 3521 compounds [98]. Such expansion of datasets will improve the accuracy and coverage of in silico models. Accurate assessment of a drug molecule’s ability to cross the BBB is determined by in vivo measurements. It has been established that Kp,uu data measured in preclinical animal studies predict human brain availability. A threshold of 0.3 for in vivo Kp,uu of mice and rats is used to estimate good human brain exposure. Moreover, there is a good correlation between animal Kp,uu data and in vitro MDR1-MDCK efflux data, which represents a valid assay for the prediction of human P-gp efflux, although other species-specific transporters may cause a disconnect. A recent publication of the physiologically based pharmacokinetic (PBPK) model shows that in vitro efflux data can be used to predict the degree of brain penetration across species accurately.

Recent efforts to develop in silico models to predict CNS penetration of small molecules focused on applying machine and deep learning methods using animal Kp,uu, and in vitro MDR1-MDCK efflux data have been less successful. Available Kp,uu data are still limited, and in silico models to predict these data with high accuracy have not yet been achieved. In addition to smaller datasets, higher variability of the measured Kp,uu values that are influenced by multiple factors such as pharmacokinetics and transporters appear to limit the accuracy of published models. Better accuracy in predicting in vitro efflux data is achieved by in silico models that utilized accurately determined experimental data on the larger sets of compounds. These in silico models are widely utilized currently to optimize lead molecules for better CNS penetration. Different scoring methods like the BBB Score have become indispensable for assessing virtual libraries for CNS targets. This review emphasizes the need for more in vitro and in vivo data to improve the accuracy of the in silico models. The forthcoming advances in data generation and modeling methods will enable the speedy and cost-effective discovery of CNS drugs.

## Figures and Tables

**Table 1:** Mean (Range) of physical–chemical properties of CNS and non-CNS drugs (copied from Pajouhesh et al. [12]).

Physical Chemical Properties	CNS	Non-CNS
Molecular weight	319 (151–655)	330 (163–671)
ClogP	3.43 * (0.16–6.59)	2.78 * (−2.81–6.09)
ClogD	2.08 (−1.34–6.57)	1.07 (−2.81–5.53)
PSA	40.5 (4.63–108)	56.1 (3.25–151)
Hydrogen bond donors	0.85 * (0–3)	1.56 * (0–6)
Hydrogen bond acceptors	3.56 (1–10)	4.51 (1–11)
Flexibility (rotatable bonds)	1.27 * (0–5)	2.18 * (0–4)
Aromatic rings	1.92 (0–4)	1.93 (0–4)

* Statistically different.
